# Extremely Efficient DFB Lasers with Flat-Top Intra-Cavity Power Distribution in Highly Erbium-Doped Fibers

**DOI:** 10.3390/s23031398

**Published:** 2023-01-26

**Authors:** Amirhossein Tehranchi, Raman Kashyap

**Affiliations:** 1 Department of Electrical Engineering, Polytechnique Montreal, University of Montreal, Montreal, QC H3C 3A7, Canada; 2 Department of Engineering Physics, Polytechnique Montreal, University of Montreal, Montreal, QC H3C 3A7, Canada

**Keywords:** distributed feedback laser, fiber laser, fiber Bragg grating

## Abstract

High-performance erbium-doped DFB fiber lasers are presently required for several sensing applications, whilst the current efficiency record is only a few percent. Additionally, a flat-top intra-cavity power distribution that is not provided in traditional DFB lasers is preferred. Moreover, cavity lengths of <20 cm are attractive for fabrication and packaging. These goals can be achieved using highly erbium-doped fiber (i.e., 110 dB/m absorption at 1530 nm), providing high gain with proper engineering of coupling coefficients. In this paper, for a given background fiber loss, first the optimum intra-cavity signal powers for various pump powers are numerically calculated. Then, for a fully unidirectional laser, optimum coupling profiles are determined. Design diagrams, including contour maps for optimum cavity lengths, maximum output powers, maximum intra-cavity signal powers, and quality factors considering various pump powers and background fiber losses, are presented. The laser pump and intra-cavity signal distribution are also calculated for a realistic, feasible modified coupling profile considering a strong unidirectionality. The DFB laser is finally simulated using generalized coupled-mode equations for such modified profiles. The efficiency of more than 22% can be realized, which is the highest reported for DFB lasers based only on erbium-doped fiber.

## 1. Introduction

The distributed feedback (DFB) fiber laser consists of a Bragg grating with a π-phase shift (PPS) written in an active fiber [1] and exhibits higher-order mode suppression as well as stable single-frequency operation [2,3,4,5,6,7,8,9,10,11,12,13,14,15,16,17,18,19,20,21,22,23,24,25,26,27,28,29,30,31,32,33,34,35]. Such π-phase-shifted gratings, initially used for DFBs in semiconductors [34], have also been implemented in erbium-doped fiber (EDF) DFB lasers [2,3,4,5,6,7,8,9]; however, the efficiency is still limited, whilst highly efficient unidirectional EDF-DFB lasers around 1.55 μm are of great importance for sensor technology, metrology, and spectroscopy amongst other applications [10,11,12,13,14]. To increase the efficiency of EDF-DFB lasers, co-doping of the fiber with ytterbium has already been used. This keeps the efficiency high for such lasers [15,16,17], however, resulting in some disadvantages. Co-doping with ytterbium ions can lead to the amplified spontaneous emission (ASE), causing a major decrease of the laser efficiency and unwanted lasing around 1 μm [18,19,20,21]. Moreover, DFB fiber lasers formed in co-doped fibers reveal much higher frequency noise [14]. Therefore, an alternative is the use of EDFs with high-concentration in the core to increase the gain [22,23,24,25,26,27]. Nevertheless, the efficiency and unidirectionality of highly EDF-DFB lasers are still restricted due to the use of conventional designs as follows. First, DFB fiber lasers have been typically realized for constant-coupling fiber Bragg grating (FBG) with a small efficiency in which a significant part of the pump power is unused [8,9]. Second, by pumping a DFB, e.g., on the near side (NS), the dislocation of the PPS from the center of the grating to the far side (FS), results in enhanced unidirectionality, as a main part of the laser power leaves the FS; however, increased unidirectionality decreases efficiency [5,28,29]. Although, the grating’s coupling profiles resulting in maximum efficiency and unidirectionality in DFB fiber lasers have been reported in [8,9,30,31,32], optimizing such profiles in a highly EDF-DFB laser considering the role of background loss has not yet been addressed. Therefore, to maximize such laser performance, engineering the realistic coupling-coefficient profiles, including the PPS positions for a given fiber background loss, is essential. The coupling coefficient as a function of length can be interpreted by considering a refractive index as a function of length and written in an EDF core.

In this paper, to maximize the laser efficiency and unidirectionality, an optimum coupling coefficient for highly EDF-DFB lasers is designed in Section 2. This is achieved with the assumption of a fully unidirectional laser resulting in a flat intra-cavity power distribution. Later, we present design diagrams to better understand the performance of EDF-DFB lasers for the combinations of design pump power and fiber background loss. In Section 3, we propose a realistic coupling profile before the PPS location considering a highly unidirectional laser. The generalized coupled-mode equations are finally used by applying the modified coupling-coefficient functions for various fiber background losses to evaluate the laser’s performance in terms of efficiency.

## 2. Ideal DFB Design for Full Unidirectionality

**Figure 1:**
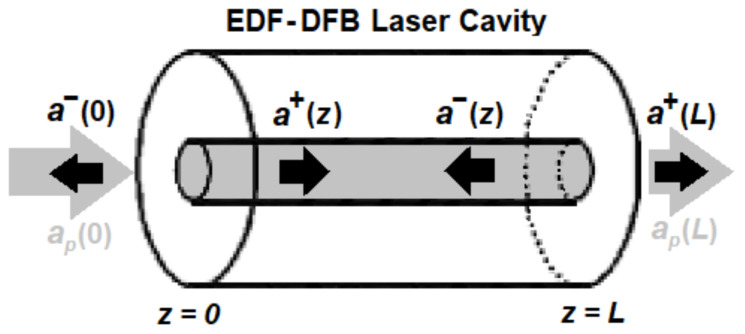
Schematic of propagating pump and signal in an EDF-DFB laser with an FBG cavity length of *L*. $a_{p}$ and (*a*^+^, *a*^−^) are the pump and signal amplitudes, respectively.

An EDF-DFB is made of an FBG with a coupling-coefficient function, $\kappa \left(z\right)$ inscribed within an erbium-doped fiber from z=0 to z=L, where L is the fiber length. For a sinusoidally varying refractive-index modulation, $\kappa \left(z\right)=\pi \Delta n\left(z\right)/\lambda_{s}$ where $\Delta n\left(z\right)$ is the amplitude of the refractive-index modulation in the grating and $\lambda_{s}$ is the laser (signal) wavelength [1]. The grating thus needs a period of $\lambda_{s}/2n_{eff}$ where $n_{eff}$ is the effective refractive index at $\lambda_{s}$. For the DFB to lase, the pump is placed at a shorter wavelength, $\lambda_{p}$, to provide the proper gain. The schematic of propagating pump and signal in an EDF-DFB laser is depicted in Figure 1.

The coupled-mode equations for the forward and backward electric field amplitudes ($a^{+}$ and $a^{-}$) in a uniform FBG under slowly varying envelopes approximation and in the steady state are expressed as [1,31]

(1) $$\frac{da^{+}\left(z\right)}{dz}=\kappa \left(z\right)a^{-}\left(z\right)+g\left(z\right)a^{+}\left(z\right),$$

(2) $$\frac{da^{-}\left(z\right)}{dz}=\kappa \left(z\right)a^{+}\left(z\right)-g\left(z\right)a^{-}\left(z\right),$$

where $g\left(z\right)$ is the gain function. The sum function, $S\left(z\right)$, and difference function, $D\left(z\right)$, can be introduced as [31]

(3) $$S\left(z\right)=a^{+}{\left(z\right)}^{2}+a^{-}{\left(z\right)}^{2},$$

(4) $$D\left(z\right)=a^{+}{\left(z\right)}^{2}-a^{-}{\left(z\right)}^{2}.$$

Using Equations (1)–(4), *D*(*z*) and $\kappa \left(z\right)$ can be written as follows, defined in [30]

(5) $$D\left(z\right)=D\left(0\right)+2\int_{0}^{z}g\left(z\prime \right)S\left(z\prime \right)dz\prime ,$$

(6) $$\kappa \left(z\right)=\left[\frac{1}{2}\frac{dS\left(z\right)}{dz}-D\left(z\right)g\left(z\right)\right]/\sqrt{S{\left(z\right)}^{2}-D{\left(z\right)}^{2},}$$

where $D\left(0\right)$ is found from Equation (4) considering the prevailing boundary-conditions. To achieve maximum output power, $S\left(z\right)$ should be maximized along the EDF. The boundary condition at *z* = 0 requires that $a^{+}\left(0\right)=0$, and $D\left(0\right)=-S\left(0\right)$. Likewise, at *z = L*, it necessitates that $a^{-}\left(L\right)=0$ and $D\left(L\right)=S\left(L\right)$ [31].

The coupled-mode equations govern the depletion of the pump power, $P_{p}$, and the amplification of the signal power, $P_{S}$, in an EDF [31,35]

(7) $$\frac{dP_{p}\left(z\right)}{dz}=-\left(d_{p}+\alpha_{p}\right)P_{p},$$

(8) $$\frac{dP_{S}\left(z\right)}{dz}=(g_{s}-\alpha_{s})P_{S},$$

where $\alpha_{p}$ and $\alpha_{s}$ are the background fiber loss for the pump and signal, respectively. Additionally, $d_{p}$ and $g_{s}$ are the pump and signal modified gain functions, respectively, as described in [9,36]

(9) $$d_{p}\left(z\right)=-\frac{\sigma_{pa}N}{w}\left\{\left(1-\frac{u}{w}\right)\ln(\frac{1+w}{1+we^{-r_{c}^{2}/\Omega_{p}^{2}}})+u\left(1-e^{-r_{c}^{2}/\Omega_{p}^{2}}\right)\right\},$$

(10) $$g_{s}\left(z\right)=\frac{\sigma_{sa}N}{w}\left\{-\left(1+\frac{v}{w}\right)\ln(\frac{1+w}{1+we^{-r_{c}^{2}/\Omega_{s}^{2}}})+v\left(1-e^{-r_{c}^{2}/\Omega_{s}^{2}}\right)\right\},$$

where $u=[\eta_{s}/\left(\eta_{s}+1\right)]\left(P_{s}{}^{+}+P_{s}{}^{-}\right)/P_{s0}$, $v=\eta_{s}P_{p}\left(z\right)/P_{p0}$ and $w=\left[P_{p}\left(z\right)/P_{p0}\right]+\left[\left(P_{s}{}^{+}+P_{s}{}^{-}\right)/P_{s0}\right]$. $P_{p0,\;s0}=\pi \Omega_{p,\;s}^{2}\;I_{p0,\;s0}$ is the threshold pump or signal power, where $I_{p0}=h\nu_{p}/\left(\sigma_{pa}t_{sp}\right)$ is the threshold pump intensity, $I_{s0}=h\nu_{s}/\left[\left(\sigma_{sa}+\sigma_{se}\right)t_{sp})\right]$ is the threshold signal intensity, $\sigma_{pa}$ is the pump absorption cross section, $\sigma_{sa}$ is the signal absorption cross section, $\sigma_{se}$ is the signal emission cross section, $\eta_{s}=\sigma_{se}/\sigma_{sa}$, $t_{sp}$ is the spontaneous emission lifetime, N is the concentration, and h is the Planck constant. $\Omega_{p,\;s}=r_{c}J_{0}\left(U\right)V\;K_{1}\left(W\right)/UK_{1}\left(W\right)$ is found for a single-mode fiber [9,36] where $U=r_{c}{\left(k_{0}^{2}n_{c}^{2}-\beta^{2}\right)}^{1/2}$,$\;V=k_{0}r_{c}{\left(n_{c}^{2}-n_{l}^{2}\right)}^{1/2},\;W=r_{c}{\left(\beta^{2}-k_{0}^{2}n_{l}^{2}\right)}^{1/2}$, and $V^{2}=U^{2}+W^{2}$. β is the propagation constant of the mode, $n_{c}$ and $n_{l}$ are the core and cladding refractive indices, $r_{c}$ is the core radius, and $k_{0}=2\pi /\lambda_{p,\;s}$ where $\lambda_{p,\;s}$ is the wavelength of EDF pump or signal. Considering the Liekki fiber, Er-110 [37], the absorptions at $\lambda_{p}=980\;\mathrm{nm}$ and $\lambda_{s}=1530\;\mathrm{nm}$ are 67 and 110 dB/m, respectively. In addition, $\alpha_{p}=\alpha_{s}$ is assumed to be in the range of 0.1–0.5 dB/m [12].

**Figure 2:**
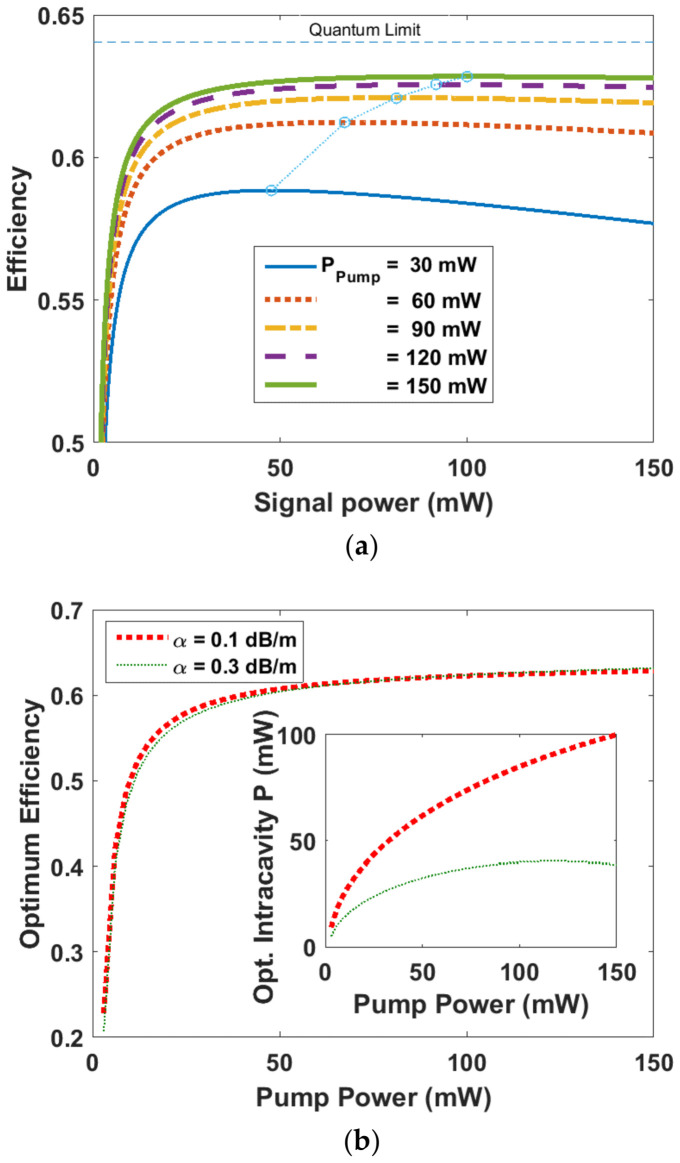
(**a**) Efficiency vs. signal power for various pump powers considering a loss of 0.1 dB/m. Blue dotted line connects the circles representing the maximum efficiencies for each curve. (**b**) Optimum efficiency vs. pump power for two amounts of loss. The inset shows the optimum intra-cavity signal power vs. pump power.

Using Equations (7)–(10) for a given pump power, by changing the signal power, a maximum intra-cavity signal power can be determined leading to find the efficiency, i.e., signal-to-pump power ratio [31]. For different pump powers, the efficiencies are calculated and shown in Figure 2a. For a typical background loss of 0.1 dB/m, the maximum ratio is always less than the quantum efficiency limit. From each peak in Figure 2a shown by circles, the maximum efficiency and optimum intra-cavity signal power for each pump power can be found. Consequently, the maximum efficiency and optimum intra-cavity signal power versus pump powers up to 150 mW have been depicted in Figure 2b for a background loss of 0.1 and 0.3 dB/m.

To engineer the coupling coefficient of the grating for the optimum efficiency of the EDF-DFB laser, Equation (6) is used. For this purpose, *D*(*z*) is needed, which is calculated using Equation (5) from the step-by-step calculation of *S*(*z*) [31]. For a given background loss and an input pump power, an optimum signal power, $P_{S}\left(0\right)$ for $P_{p}\left(0\right)$ is found (e.g., using the data in Figure 2b). The pump at z=δz is $P_{p}\left(\delta z\right)=P_{p}\left(0\right)+\delta P_{p}\left(0\right)\delta z$ where $\delta P_{p}\left(0\right)=-d_{p}P_{S}\left(0\right)P_{p}\left(0\right)-\alpha_{p}P_{p}\left(0\right)$. We then find $P_{S}\left(\delta z\right)$ for $P_{p}\left(\delta z\right)$ (e.g., using the data in Figure 2b) and calculate likewise $P_{p}\left(2\delta z\right)$ and $P_{S}\left(2\delta z\right)$. Consequently, the pump and signal power distributions over the entire EDF-DFB length can be calculated. To compute $D\left(z\right)$, $D\left(0\right)$ is needed. Here, co-directional signal and pump in a fully unidirectional laser with $a^{-}\left(0\right)=0$, results in $D\left(0\right)=0$.

**Figure 3:**
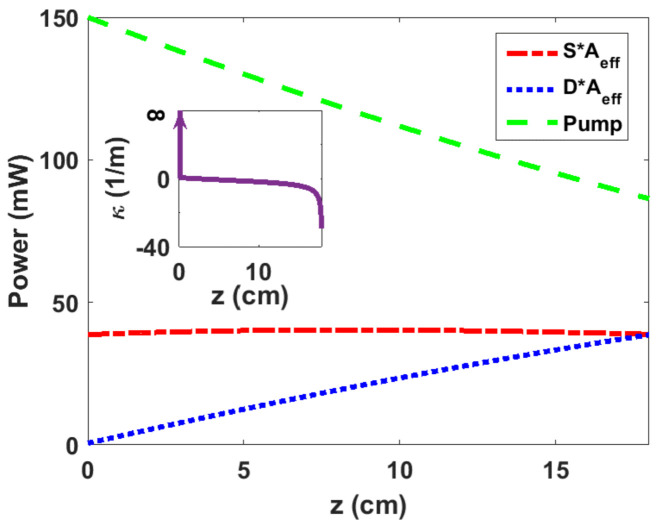
Distribution of *SA_eff_* and *DA_eff_* and *P_p_* as a function of EDF-DFB length for a pump power of 150 mW and a loss of 0.3 dB/m, resulting in *S = D* at 18 cm. Note, the intra-cavity power distribution (in red) is nearly flat. The inset shows the coupling coefficient distribution over the 18-cm-long EDF-DFB. Coupling coefficient is very large at *z* = 0 due to the full unidirectionality assumption. The zero crossing, i.e., PPS occurs at 1.9 cm.

To maximize an EDF-DFB laser efficiency considering a typical background loss of 0.3 dB/m and pump power of 150 mW, optimum *P_p_*(*z*), *D*(*z*), and *S*(*z*) are calculated based on the above-mentioned procedures. Figure 3 depicts *P_p_*, *DA_eff_*, and *SA_eff_* as a function of EDF-DFB length where $A_{eff}$ is the EDF effective mode area. *S* and *D* curves intersect at *z* = *L* = 18 cm dictated by $S\left(L\right)=D\left(L\right)$, resulting in the FS signal power of 38.6 mW. The intra-cavity power versus the length is nearly flat, and its maximum reaches almost 40.3 mW. The design thus enables the realization of a DFB laser for which the intra-cavity power distribution is flat top, and its peak is close to the output power. A flat-top distribution for intra-cavity power which is not provided in traditional DFB designs is preferred for some sensing applications due to enhanced effective cavity length [12,31]. The optimized EDF-DFB fiber laser then consists of an FBG with an optimized length of 18 cm and an optimized coupling function, $\kappa \left(z\right)$, which is calculated by inserting $S\left(z\right)$ and $D\left(z\right)$ in Equation (6). The inset in Figure 3 illustrates the coupling coefficient as a function of EDF-DFB length. The sign change in $\kappa \left(z\right)$ is achieved by applying a PPS [28].

**Figure 4:**
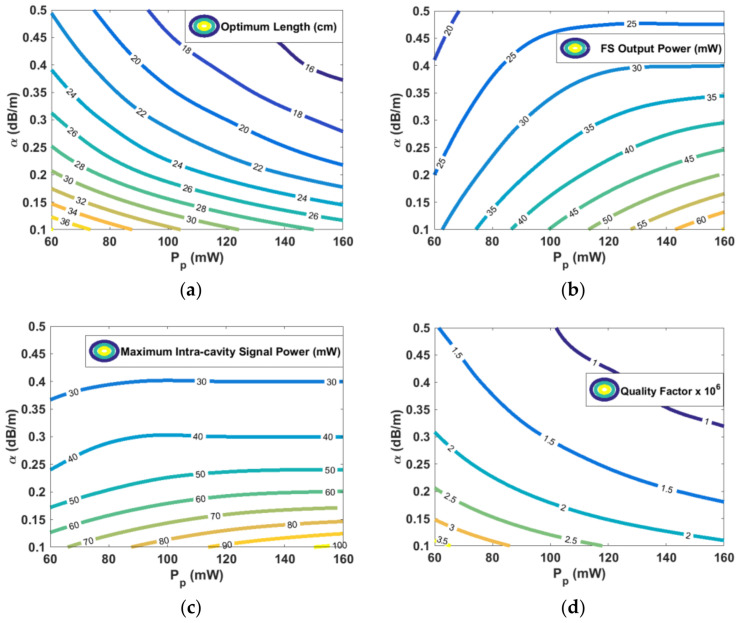
Contour maps for (**a**) optimum cavity length, (**b**) maximum FS output power, (**c**) maximum intra-cavity signal power, and (**d**) quality factor, considering various pump power and fiber background loss.

It is worth noting as the main parameters, including the optimum DFB length, FS output power, maximum intra-cavity power, and quality factor, are functions of the pump power and fiber background loss. Therefore, the aforesaid parameters are plotted as contour plots to provide useful design diagrams. Figure 4a shows optimum length versus the background loss and power. It is evident that for a constant pump power, a lower loss results in a longer optimum length. Figure 4b depicts fully unidirectional output power versus the background loss and power. It is obvious that for a constant pump power, a lower loss results in a larger output power. Figure 4c illustrates maximum intra-cavity power versus the background loss and pump power. For a constant pump power, a lower loss results in a larger intra-cavity peak power. Finally, the cavity *Q*-factor is computed using [28,33]

(11) $$Q=\frac{2\pi n_{eff}\int_{0}^{L}\left[{\left|a^{+}\left(z\right)\right|}^{2}+{\left|a^{-}\left(z\right)\right|}^{2}\right]dz}{\lambda_{s}\left[{\left|a^{+}\left(L\right)\right|}^{2}+{\left|a^{-}\left(0\right)\right|}^{2}\right]}.$$

Figure 4d plots the quality factor versus the background loss and power. It is apparent that for a constant pump power, a lower loss results in a larger *Q*-factor. It is worth mentioning, although increasing the pump power for a constant background loss leads to shorter optimum length and larger output power, as can be seen in Figure 4a,b, respectively, it also may result in larger intra-cavity peak power and smaller *Q*-factor, as can be found in Figure 4c,d, respectively.

## 3. Realistic DFB Design for Strong Unidirectionality

For the next step in the design, we must find a realistic distribution for the coupling coefficient to be feasible. As discussed formerly, to make a fully unidirectional laser, the NS signal output power must be set equal to zero so all the generated power exits the cavity from the FS. However, this condition will need a perfect mirror at the NS, making the realization hard in practice. Thus, we assume a non-zero left-hand output power and permit a gradual change for the optimum signal power before the PPS, resulting in a coupling coefficient distribution that can be realized by an FBG writing technique. In this paper, we consider the signal power distribution before the PPS to be exponential, i.e., $S_{exp}\left(z\right)=S\left(0\right)\exp\left(qz\right)$, where *q* is the exponential coefficient. The distribution of *P_p_* and *D* thus experience an exponential profile before the PPS. Based on the design diagram of Figure 4a for a zero NS output power (ideal), an optimum design length of 18 cm is found for a loss of 0.3 dB/m and a pump power of 150 mW. For a realistic design, we consider the NS output power to be as low as 10 µW; therefore, $S\left(0\right)=10\;\mu W$ and the unidirectionality (the FS-to-NS output signal power ratio) is almost 36 dB. 

The exponentially increasing signal, $S_{exp}$, encounters the optimum value for the signal power of 39.4 mW at the PPS position of 1.9 cm, and from this location, the signal power distribution is assumed to follow the optimum value. With the increase in the signal power, we calculate the pump power and *D* parameter distribution starting from 150 mW. Knowing the optimum signal power for each pump power (see Figure 2b), the optimum signal distribution corresponding to this pump distribution can be obtained.

**Figure 5:**
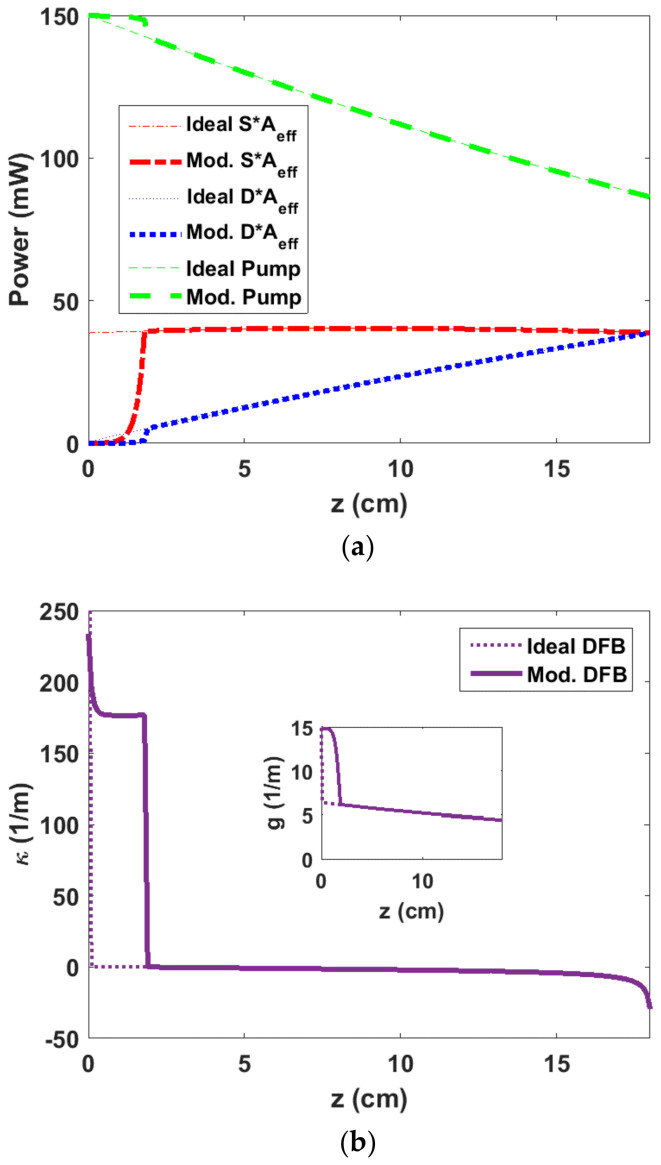
(**a**) Modified distribution of *SA_eff_* and *DA_eff_* and *P_p_* as a function of EDF-DFB length for a pump power of 150 mW and a loss of 0.3 dB/m. An exponential distribution for *S* is considered before PPS position at 1.9 cm, and the realistic non-zero NS output power is assumed to be as low as 10 µW. (**b**) Modified coupling coefficient vs. length for an 18-cm-long DFB with a maximum coupling of 234 m^−1^. The inset shows the gain vs. the DFB length.

Figure 5a shows ideal and modified curves for *P_p_*, *S*, and *D* as a function of EDF-DFB length. The exponential parts of the curves before the PPS (z = 1.9 cm) in Figure 5a depict the described modifications. With the aforementioned parameters we use Equations (5) and (6) to re-calculate $D\left(z\right)$ and $\kappa \left(z\right)$ up to the PPS position at z = 1.9 cm. Consequently, the modified DFB’s coupling-coefficient profile is shown in Figure 5b.

The calculated maximum coupling coefficient is $\kappa_{max}=234{\;m}^{-1}$, as shown in Figure 5b, corresponding to an effective refractive index modulation, $\Delta n_{max}=\frac{\kappa_{max}\lambda_{s}}{\pi }=1.1\times 10^{-4}$, for a signal wavelength at 1530 nm, which can be readily obtained with the FBG-writing technology. It is worth noting that in practice, the minimum NS output power and consequently, the maximum unidirectionality depend on the maximum coupling coefficient that can be achieved in the FBG. Furthermore, as the pump and signal distributions are known, the gain is then computed and shown in the inset of Figure 5b, representing an exponential variation before PPS for the modified DFB.

To assess the performance of the EDF-DFB laser for the modified coupling profile, the following generalized coupled-mode equations governing the evolutions of pump and signal amplitudes are used, as defined in [9]

(12) $$\frac{\partial a_{p}}{\partial z}=\left\{\frac{1}{2}\left(d_{p}-\alpha_{p}\right)+i\gamma_{p}\left[{\left|a_{p}\right|}^{2}+2{\left|a_{s}{}^{+}\right|}^{2}+2{\left|a_{s}{}^{-}\right|}^{2}\right]\right\}\;a_{p},$$

(13) $$\frac{\partial a_{s}{}^{+}}{\partial z}=\left\{\frac{1}{2}\left(g_{s}-\alpha_{s}\right)-i\left[-\gamma_{s}\left(2{\left|a_{p}\right|}^{2}+{\left|a_{s}{}^{+}\right|}^{2}+2{\left|a_{s}{}^{-}\right|}^{2}\right)-\xi \right]\right\}a_{s}{}^{+}+i\kappa a_{s}{}^{-},$$

(14) $$-\frac{\partial a_{s}{}^{-}}{\partial z}=\left\{\frac{1}{2}\left(g_{s}-\alpha_{s}\right)-i\left[-\gamma_{s}\left(2{\left|a_{p}\right|}^{2}+2{\left|a_{s}{}^{+}\right|}^{2}+{\left|a_{s}{}^{-}\right|}^{2}\right)-\xi \right]\right\}a_{s}{}^{-}+i\kappa a_{s}{}^{+},$$

where ${\left|a_{s}{}^{\pm }\right|}^{2}=P_{s}{}^{\pm }$ and ${\left|a_{p}\right|}^{2}=P_{p}$. The nonlinear coefficients for the signal and pump are $\gamma_{s}=2\pi n_{2}/\lambda_{s}A_{eff}$ and $\gamma_{p}=\gamma_{s}\;\lambda_{s}/\lambda_{p}$, respectively, where the nonlinear refractive index is $n_{2}=2.2\times 10^{-20}{\;m}^{2}/W$. The signal-detuning parameter is $\xi =n\left(\omega -\omega_{s}\right)/c$ where *n* is the refractive index of the EDF core, ω is the lasing frequency, and *c* is the light speed in vacuum. For the assessment of EDF-DFB laser efficiency, we solve the Equations (12)–(14) using the Runge–Kutta method [9,28] assuming the EDF parameters already utilized in Section 2 and considering the modified $\kappa \left(z\right)$.

As an example, we consider designing 18-cm-long DFB fiber lasers using the design diagram for optimum lengths. Based on Figure 4a, for three given losses of 0.1, 0.3, and 0.5 dB/m, there are three design pump powers of 440, 150, and 90 mW, respectively, and consequently, three different design coupling-profiles, $\kappa \left(z\right)$. 

**Figure 6:**
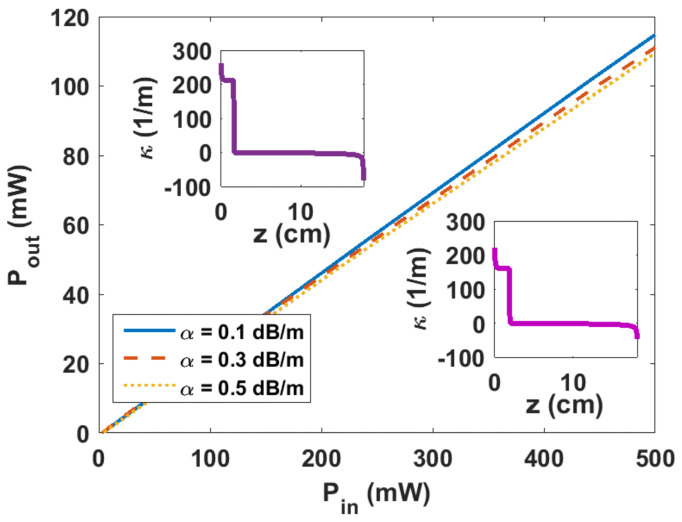
Output power vs. input pump power for three designs considering three various loss parameters of 0.1, 0.3, and 0.5 dB/m and the same optimum length of 18 cm. The insets show the modified coupling coefficient distributions vs. the 18-cm-long DFB for a loss of (top) 0.1 dB/m and (down) 0.5 dB/m for which (maximum coupling-coefficient, PPS position) are (261 m^−1^, 1.7 cm) and (221 m^−1^, 2 cm), respectively. For a loss of 0.3 dB/m, $\kappa \left(z\right)$ has been shown in Figure 5b.

It is worth noting that by decreasing the design pump powers (440, 150, and 90 mW), the maximum coupling coefficients are decreased (261, 234, and 221 m^−1^), whilst the PPS positions are increased (1.7, 1.9, and 2 cm). Solving Equations (12)–(14), we calculate the output power versus various pump powers up to 500 mW, as depicted in Figure 6, considering the three coupling-profiles for different losses which are shown in Figure 5b and the insets of Figure 6. For given losses of 0.1, 0.3, and 0.5 dB/m, at the design pump powers, the DFB output powers are 101.3 mW, 34.1 mW, and 19.4 mW, and the DFB lasers have linear output/input power characteristics with the slopes of 23.1%, 22.3%, and 22.1%, respectively.

## 4. Conclusions

The design of extremely efficient and unidirectional DFB lasers in highly doped-erbium fiber based on the tailoring of the FBG-coupling-coefficient profile in the presence of various fiber background losses has been proposed. For a given pump power, the FBG’s optimum coupling function for fully unidirectional laser with flat-top intra-cavity power distribution was found. To evaluate optimum FBG length, maximum output power, maximum intra-cavity signal power, and quality factor and considering various pump power and fiber background loss, design diagrams, including contour maps, are presented. The laser design was then modified for a realistic coupling profile considering a strong unidirectionality, which can be readily controlled by adjusting the maximum coupling coefficient. The EDF-DFB laser was finally simulated using generalized coupled-mode equations for such modified profiles. As an example, the short 18-cm-long FBG designs enable fabrication of the highly efficient EDF-DFB laser with the efficiency more than 22%, the highest value reported for the lasers based only on erbium-doped fiber. Although here, the background fiber loss and the doping concentrations of EDF are assumed constant along the fiber length, our model is capable of considering them as functions of length for possible longitudinal variations, including non-uniformities. Our approach shows different DFB lengths and background losses can be accommodated to provide optimum output power.

## Data Availability

The data presented in this study are available on request from the corresponding author.
